# Sepsis in Patients With Large Vessel Occlusion Stroke–Clinical Characteristics and Outcome

**DOI:** 10.3389/fneur.2022.902809

**Published:** 2022-07-12

**Authors:** Sebastian Stösser, Julia Isakeit, Felix J. Bode, Christian Bode, Gabor C. Petzold

**Affiliations:** ^1^Division of Vascular Neurology, Department of Neurology, University Hospital Bonn, Bonn, Germany; ^2^Department of Anesthesiology and Intensive Care Medicine, University Hospital Bonn, Bonn, Germany

**Keywords:** sepsis, ischemic stroke, thrombectomy, patient outcome assessment, infections, organ dysfunction scores

## Abstract

**Background:**

Infections are an important complication after stroke and negatively affect clinical outcome. While pneumonia and urinary tract infections are well recognized after stroke, the incidence and consequences of sepsis remain unclear. The aim of this study was to evaluate the frequency and characteristics of sepsis in patients undergoing endovascular therapy for large vessel occlusion stroke, and its association with clinical outcome.

**Methods:**

We analyzed a cohort of patients who underwent endovascular therapy at a single center between 2016 and 2020. The diagnosis and timing of infections and Sequential Organ Failure Assessment scores were evaluated retrospectively to identify patients with sepsis. Patients with sepsis were compared to controls regarding clinical characteristics and outcome.

**Results:**

Fifty-four of 406 patients (13.3%) were found to have sepsis. The median onset of sepsis was 2 days after admission. The majority of cases (85.2%) was caused by pneumonia. At 3 months, 72.5% of patients with sepsis were bedridden or dead compared to 25.7 and 42.7% of controls and patients with an infection without sepsis, respectively. The adjusted odds ratio (95% confidence interval) for a poor outcome was 5.4 (1.6–17.6) for patients with sepsis vs. controls, and 2.0 (0.8–5.2) for patients with sepsis vs. patients with an infection without sepsis.

**Conclusions:**

Sepsis is a frequent complication after large vessel occlusion stroke, and may be associated with a poor clinical outcome. More studies are needed to determine specific risk factors and measures to early recognize and reduce the possibly negative impact of sepsis on outcome after stroke.

## Introduction

Infections are an important complication after stroke and have a strong association with an unfavorable clinical outcome (1, 2). The most common stroke-associated infections are pneumonia and urinary tract infections, occurring in 12 and 8–19%, respectively (3, 4). While these infections are well recognized after stroke, less is known about sepsis after stroke. Berger et al. reported sepsis in 12.6% of patients admitted to a neurological intensive care unit for ischemic or hemorrhagic stroke and an association of sepsis with poor outcome in 2014 (5).

In 2016, new diagnostic criteria for sepsis were established. The Sepsis-3 definition relies on the detection of life-threatening organ dysfunction consequent to an infection assessed by the Sequential Organ Failure Assessment (SOFA) score (acute increase of ≥2 points) (6). Further, the quick Sequential Organ Failure Assessment (qSOFA) score was introduced to facilitate the diagnosis of sepsis in a non-intensive care unit setting (7). However, studies employing the Sepsis-3 definition to investigate sepsis after ischemic stroke are missing.

The aim of this study was to evaluate the frequency and characteristics of sepsis in patients undergoing endovascular therapy (ET) for LVOS using the Sepsis-3 definition, to evaluate the impact of sepsis on clinical outcome in these patients, and to assess the predictive value of the qSOFA score.

## Methods

All data analyzed in this study were derived from patients included in the German Stroke Registry–Endovascular Therapy (GSR-ET) Study at University Hospital Bonn between June 2016 and January 2020. The GSR-ET is an ongoing, open-label, prospective, multicenter registry of patients with LVOS treated with ET (https://www.clinicaltrials.gov; unique identifier: NCT0335639). A detailed description of the GSR-ET study design has been published (8, 9). Data collection was centrally approved by the ethics committee of the Ludwig-Maximilian University Munich (689-15) and the local ethics committee of the University of Bonn (054/16) and was thus in accordance with the ethical standards laid down in the 1964 Declaration of Helsinki and its later amendments. The data supporting the findings of this study are available from the corresponding author upon reasonable request.

In addition to data prospectively collected in GSR-ET, clinical and laboratory data were retrospectively collected from individual electronic medical records. Patients with infections were identified by screening medical records for, firstly, a diagnosis of infection documented by the treating clinician and, secondly, subsequent antibiotic therapy. Diagnosis and treatment of infections was in accordance with current national guidelines (10–12). A summary of the standard operating procedures for diagnosis and treatment of infections at our institution is provided in the supplement. Pneumonia was also evaluated according to the modified Centers for Disease Control and Prevention (CDC) criteria for probable stroke-associated pneumonia and according to the CDC criteria for health care-associated pneumonia in ventilated patients (13, 14). The source of infections was classified as “other” if there was a clear source of infection other than pneumonia or urinary tract infection and as “undetermined” if the source of infection was unclear after diagnostic workup. Patients who were diagnosed with an infection, but not treated by antibiotics because of a comfort measures only agreement, were defined as having an infection too. The onset of infection was defined as the first day of either antibiotic therapy or microbiological culture sampling (if sampling was done within 48 h prior to antibiotic therapy) (7). SOFA and qSOFA scores were determined at admission and daily within the period from 2 days before the onset of infection to 1 day after the onset of infection (7, 15). The SOFA score assesses the function of six vital organ systems based on physiological parameters (15). The qSOFA score is a simplified version of the SOFA score developed to screen patients with an infection for sepsis outside the intensive care unit (7). Patients who did not have an infection served as controls. In this group, SOFA and qSOFA scores were determined at admission and for the following 4 days. If P_a_O_2_ was not available, the P_a_O_2_/FiO_2_ ratio was substituted by the SpO_2_/FiO_2_ ratio as described previously (16). Sepsis was defined as an increase in total SOFA score of two points or more over the baseline score at admission within the period from 2 days before the onset of infection to 1 day after the onset of infection according to the Sepsis-3 definition (6).

Primary outcome measure was the frequency of patients with a score of five to six on the modified Rankin Scale (mRS) after 90 days. Secondary outcome measures included National Institutes of Health Stroke Scale (NIHSS) scores, the length of hospitalization, the frequency of patients with a mRS score of zero to two, median mRS scores and mortality. Symptomatic intracranial hemorrhage was evaluated retrospectively according to the European Cooperative Acute Stroke Study (ECASS) II definition (17).

Statistical analyses were performed using the Statistical Package for Social Sciences version 27.0.0.0 (IBM SPSS Statistics, Armonk, N.Y., USA). Propensity score matching was performed using R (R version 4.1.3, R core team 2021, package “MatchIt”, R Foundation for Statistical Computing, Vienna, Austria. https://www.R-project.org/). Propensity score matching was performed using a 1:1 nearest neighbor matching without replacement. Propensity scores were estimated using logistic regression with the following relevant covariates: age, sex, NIHSS at admission, premorbid mRS and Charlson Comorbidity Index. Cases with missing data on those covariates were excluded from the analysis of matched cohorts. Differences in metric data were assessed using Kruskal-Wallis tests and Mann-Whitney U tests where appropriate. Differences in frequencies were assessed using Pearson chi-square tests. Regression analyses were conducted using multivariable linear, logistic and ordinal models based on the dependent variable. Multivariable analyses of outcome measures were adjusted for a predefined set of variables (age, sex, NIHSS at admission, premorbid mRS, Charlson Comorbidity Index, and either intracranial hemorrhage for the 24 h and discharge follow ups, or the combined frequency of intracranial hemorrhage, recurrent stroke and malignant infarction within 90 days for the 90 days follow up). Missing SOFA score variables from the day of admission were imputed by the value from the following day (“carried backwards”), all other missing data including follow up SOFA score variables were not imputed. Sensitivity and specificity were calculated using standard formula. All tests were two-tailed. Statistical significance was determined at an α level of 0.05. A Bonferroni-Holm correction was applied to *p*-values for pairwise comparisons.

## Results

The data of 406 patients who underwent ET for LVOS between June 2016 and December 2019 were available and included in the analysis. Fifty-four (13.3%) patients had sepsis. One hundred fifty-eight (38.9%) patients had an infection without fulfilling the Sepsis-3 definition. The remaining 194 (47.8%) patients had no evidence of infection (see Supplementary Figure S1 for the study flowchart).

The demographics, medical history as well as clinical, imaging and treatment characteristics of patients with sepsis, patients with an infection without sepsis, and control patients are shown in Table 1. The three cohorts were evenly balanced regarding these baseline variables with the following significant exceptions. Patients with an infection without sepsis were older than controls (median 79 vs. 74 years). Patients with sepsis and patients with an infection without sepsis had a higher frequency of arterial hypertension compared to controls (90.7 and 87.3, respectively, vs. 76.4%). Patients with an infection without sepsis had a higher frequency of atrial fibrillation compared to controls (54.5 vs. 41.1%).

**Table 1:** Baseline characteristics of control patients, patients with an infection without sepsis and patients with sepsis.

	**Controls** **% (*n/N*)**	**Infection without sepsis** **% (*n/N*)**	**Sepsis** **% (*n/N*)**
Age, year, median (Q1–Q3) *n*, cases available	74 (63–82) *n* = 194	79 (69–84)^*^ n = 158	76 (64–83) *n* = 54
Sex, female	57.7% (112/194)	55.7% (88/158)	42.6% (23/54)
Arterial hypertension	76.4% (146/191)	87.3% (137/157)^*^	90.7% (49/54)^†^
Dyslipidemia	67.2% (129/192)	65.6% (103/157)	50.9% (27/53)
Atrial fibrillation	41.1% (78/190)	54.5% (85/156)^*^	46.2% (24/52)
Smoking	19.4% (36/186)	12.2% (18/147)	20.8% (10/48)
Diabetes mellitus	21.1% (44/190)	23.1% (36/156)	18.9% (10/53)
Charlson comorbidity index score, median (Q1–Q3)	1 (0–2) *n* = 194	1 (0–2) *n* = 158	1 (0–3) *n* = 54
Premorbid modified rankin scale score, median (Q1–Q3)	0 (0–1) *n* = 188	0 (0–2) *n* = 152	0 (0–1) *n* = 51
Onset of symptoms known	57.7% (112/194)	48.7% (77/158)	63.0% (34/54)
Time from onset to admission, min, median (Q1–Q3)	105 (55–198) *n* = 194	95 (57–194) *n* = 158	89 (60–220) *n* = 54
ASPECTS at admission	8 (8–10) *n* = 154	8 (7–9) *n* = 77	8 (7–10) *n* = 35
NIHSS score at admission	13 (9–17) *n* = 191	14 (10–17) *n* = 156	14 (11–17) *n* = 52
Occluded vessel			
Middle cerebral artery, M1 segment	60.5% (115/190)	53.5% (84/157)	48.1% (26/54)
Middle cerebral artery, M2 segment	20.5% (39/190)	20.4% (32/157)	25.9% (14/54)
Intracranial internal carotid artery	18.4% (35/190)	22.9% (36/157)	18.5% (10/54)
Basilar artery	10.0% (19/190)	8.9% (14/157)	14.8% (8/54)
Other	1.6% (3/190)	1.9% (3/157)	7.4% (4/54)
Side of occluded vessel, left	52.9% (91/172)	49.3% (73/148)	52.1% (25/48)
Stroke etiology			
Cardioembolism	49.2% (94/191)	60.8% (96/158)	52.8% (28/53)
Large artery arteriosclerosis	22.0% (42/191)	18.4% (29/158)	17.0% (9/53)
Other determined etiology	3.7% (7/191)	4.4% (7/158)	7.5% (4/53)
Undetermined etiology	25.1% (48/191)	16.5% (26/158)	22.6% (12/53)
Intravenous thrombolysis	53.6% (104/194)	41.8% (66/158)	46.3% (25/54)
Successful recanalization (mTICI 2b-3)	96.3% (154/160)	93.3% (111/119)	92.9% (39/42)
General anesthesia	100% (192/192)	100% (157/157)	100% (53/53)
Time from onset to flow restoration, min, median (Q1–Q3)	228 (188–314) *n* = 88	231 (192–315) *n* = 58	252 (190–352) *n* = 27

*ASPECTS, Alberta stroke programme early CT score; NIHSS, National Institutes of Health Stroke Scale; mTICI, modified Thrombolysis In Cerebral Infarction Scale*.

* *Indicates a significant difference between patients with an infection without sepsis and controls (p < 0.05 after Bonferroni-Holm adjustment for multiple comparisons)*.

† *Indicates a significant difference between sepsis patients and controls (p < 0.05 after Bonferroni-Holm adjustment for multiple comparisons)*.

Details regarding the timing and site of infection and the SOFA scores are given in Table 2. The median onset of sepsis was 2 days after admission, compared to 3 days for the median onset of infection without sepsis. Pneumonia was the most common source of sepsis (85.2%) and infection without sepsis (56.3%) and was more common in patients with sepsis than in patients without sepsis. If pneumonia was evaluated to the stricter modified CDC criteria for probable stroke-associated pneumonia, the results were similar (75.5% in patients with sepsis vs. 41.7% in patients with an infection without sepsis). The overall frequency of pneumonia in all three groups combined was 33.2% (135/406) and 25.9% (105/406) according to the modified CDC criteria. Dysphagia with risk of aspiration was more common in patients with sepsis compared to patients with an infection without sepsis and controls (74.1 vs. 39.2 and 17.9%, respectively). The same held true for mechanical ventilation (49.1 vs. 15.4 and 12.9%, respectively). The overall frequency of urinary tract infections in all three groups combined was 10.6% (43/406). Moreover, the cause of infection was undetermined in more patients with infection without sepsis (15.2%) than in patients with sepsis (3.7%).

**Table 2:** Timing, source of infection and Sequential Organ Failure Assessment (SOFA) scores of controls, patients with an infection without sepsis and patients with sepsis.

	**Controls** **% (*n/N*)**	**Infection without sepsis** **% (*n/N*)**	**Sepsis** **% (*n/N*)**
Time from admission to diagnosis of infection, days, median (Q1–Q3) *n*, available cases	–	3 (1–5) *n* = 155	2 (1–5) *n* = 53
Source of infection			
Pneumonia (clinical diagnosis)	–	56.3% (89/158)	85.2% (46/54)^‡^
Pneumonia (diagnosis according to modified CDC criteria)	–	41.7% (65/156)	75.5% (40/53)^‡^
Urinary tract infection	–	22.8% (36/158)	13.0% (7/54)
Other	–	7.0% (11/158)	1.9% (1/54)
Undetermined	–	15.2% (24/158)	3.7% (2/54)^‡^
Dysphagia with risk of aspiration	17.9% (34/190)	39.2% (62/158)^*^	74.1% (40/54)^†‡^
Mechanical ventilation on assessment of follow up SOFA scores	12.9% (24/186)	15.4% (23/149)	49.1% (26/53)^†‡^
Duration of mechanical ventilation, h, median (Q1–Q3)	0 (0–0) *n* = 24	0 (0–0) *n* = 23	0 (0–53.5)^†‡^ *n* = 26
Positive microbiological cultures	–	54.9% (50/91)	57.1% (24/42)
Positive blood cultures	–	27.6% (16/58)	33.3% (9/27)
SOFA score at admission, median (Q1–Q3)	2 (0–3) n=192	3 (1–4)* n = 157	2 (1–5) *n* = 53
Maximum SOFA score, median (Q1–Q3)	2 (1–4) n=194	3 (2–4)* n = 157	8 (4–11)^†‡^ *n* = 54
SOFA subcategories–increase of score ≥2 compared to admission			
Central nervous system	6.6% (11/166)	0.7% (1/150)^*^	37.7% (20/53)^†‡^
Respiration	7.6% (13/172)	5.3% (7/133)	53.8% (28/52)^†‡^
Coagulation	0.5% (1/194)	0% (0/156)	1.9% (1/54)
Liver	2.1% (1/48)	1.9% (1/52)	3.0% (1/33)
Cardiovascular	2.4% (4/168)	0% (0/153)	35.8% (19/53)^‡^
Renal	0% (0/194)	0.6% (1/156)	7.4% (4/54)^‡^
Increase of maximal SOFA score ≥2 without CNS subcategory	12.4% (24/194)	3.8% (6/157)	83.3% (45/54)

*CDC, Centers for Disease Control and Prevention; SOFA, Sequential Organ Failure Assessment; CNS, Central nervous system*.

* *Indicates a significant difference between patients with an infection without sepsis and controls (p < 0.05 after Bonferroni adjustment for multiple comparisons)*.

† *Indicates a significant difference between sepsis patients and controls (p < 0.05 after Bonferroni adjustment for multiple comparisons)*.

‡ *Indicates a significant difference between sepsis patients and patients with an infection without sepsis (p < 0.05 after Bonferroni adjustment for multiple comparisons)*.

The median of the maximal SOFA score within the period of 2 days before until 1 day after the onset of infection of patients with sepsis was eight (from a baseline of two), while the SOFA scores did not increase in controls and patients with an infection without sepsis as per definition. The time courses of total SOFA scores and scores of SOFA subcategories are shown in Supplementary Figure S2. The most frequently affected organ systems in patients with sepsis were respiration (53.8%), the central nervous system (37.7%), the cardiovascular system (35.8%) and renal function (7.4%). If SOFA scores were calculated without the central nervous system (CNS) subcategory, 9 (16.7%) patients with sepsis failed to meet the Sepsis-3 definition. On the other hand, 6 (3.8%) patients with an infection without sepsis did meet the Sepsis-3 definition if the CNS subcategory was left out. Additional laboratory and clinical parameters are shown in Supplementary Table S1.

The qSOFA score was significantly more often positive in patients with sepsis at the onset of infection (86.8%) compared to patients with an infection without sepsis (58.5%) and to controls (37.2%). This resulted in a sensitivity of 86.8% and a specificity of 52.2% of the qSOFA score for the diagnosis of sepsis. When analyzing the qSOFA subcategories, the mental status showed a sensitivity and specificity of 94.3 and 37.5%, respectively, the respiratory rate a sensitivity and specificity of 71.7 and 30.0%, respectively, and the systolic blood pressure a sensitivity and specificity of 49.1 and 75.6%, respectively. The data on qSOFA scores are shown in Supplementary Table S2.

**Figure 1:**
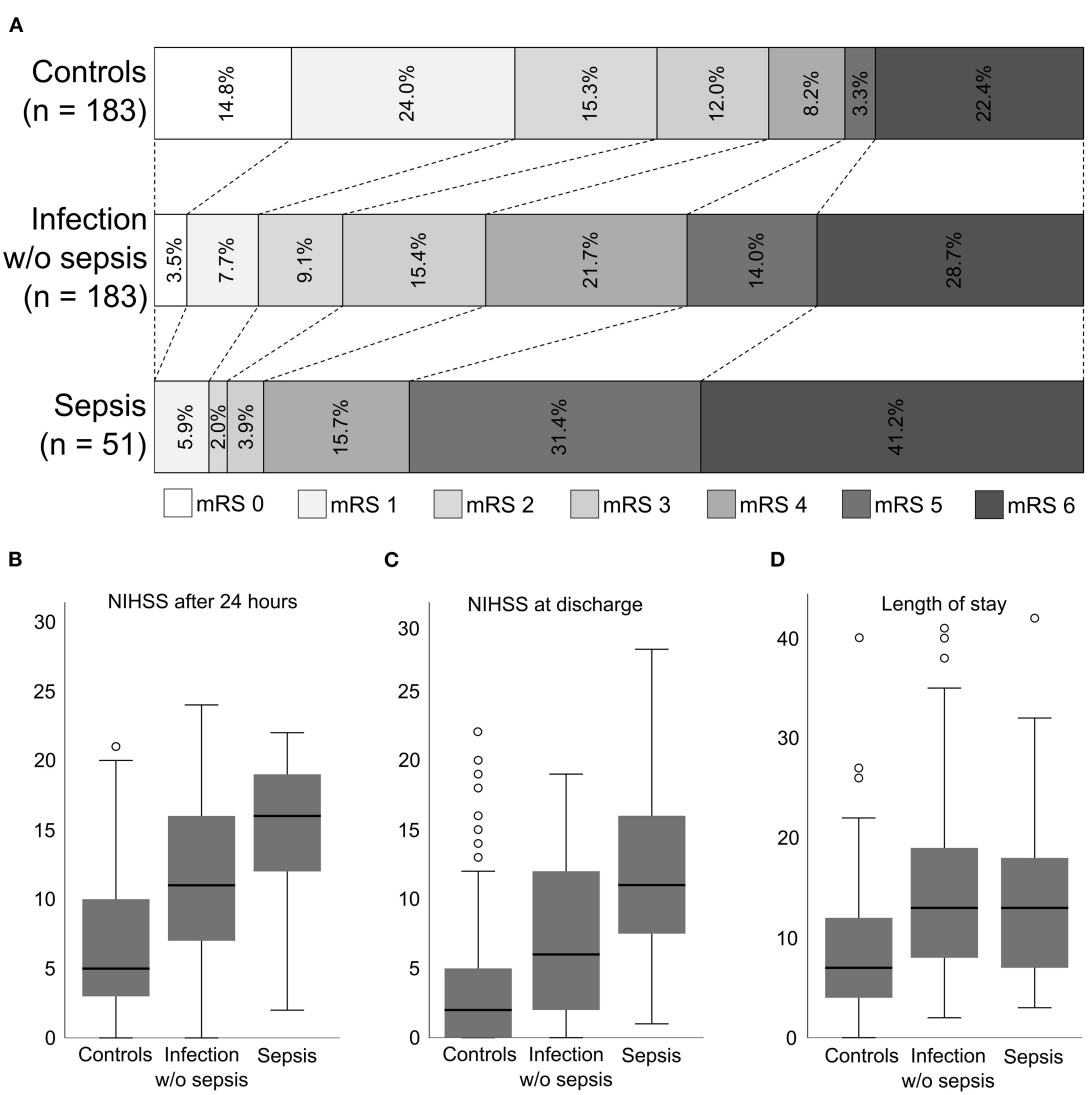
Primary and secondary outcome parameters of patients with sepsis, patients with infection without sepsis and control patients. **(A)** modified Rankin scale (mRS) scores at 3 months. **(B)** National Institutes of Health Stroke Scale (NIHSS) scores at 24 h. **(C)** NIHSS scores at hospital discharge. **(D)** Length of hospitalization in days. The data in **(B–D)** are displayed as boxplots; the box represents the median and interquartile range; the whiskers represent 1.5 times the interquartile range; the circles represent outliers.

Clinical outcome parameters are shown in Table 3 and are illustrated in Figure 1. A poor outcome at 90 days, indicated by an mRS of five or six, was more common in patients with sepsis compared to controls and to patients with an infection without sepsis (72.5 vs. 25.7 and 42.7%, respectively). The adjusted odds ratio (95% confidence interval) for a poor outcome was 11.4 (4.4–29.2) for patients with sepsis vs. controls, 3.5 (1.6–7.5) for patients with sepsis vs. patients with an infection without sepsis, and 5.9 (2.7–12.9) for patients with sepsis vs. all patients without sepsis. If adjusted for the NIHSS at 24 hours instead of NIHSS at admission, the odds ratio (95% confidence interval) for a poor outcome was 5.4 (1.6–17.6) for patients with sepsis vs. controls, 2.0 (0.8–5.2) for patients with sepsis vs. patients with an infection without sepsis, and 2.7 (1.0–6.8) for patients with sepsis vs. all patients without sepsis.

**Table 3:** Clinical outcome of controls, patients with an infection without sepsis and patients with sepsis.

	**Controls** **% (*n/N*)**	**Infection without sepsis** **% (*n/N*)**	**Sepsis** **% (*n/N*)**
**24 h follow-up**			
NIHSS, median (Q1–Q3) *n*, available cases	5 (3–10) *n* = 162	11 (7–16)^*^ n = 148	16 (12–19)^†^^‡^ *n* = 43
Any intracranial hemorrhage	4.7% (9/192)	8.3% (13/156)	11.1% (6/54)
Symptomatic intracranial hemorrhage	1.1% (2/190)	2.6% (4/156)	1.9% (1/52)
**Discharge follow up**			
NIHSS, median (Q1–Q3)	2 (0–5) *n* = 141	6 (2–12)^*^ n = 111	11 (6–17)^†^^‡^ *n* = 27
Length of stay, d, median (Q1–Q3)	7 (4–12) *n* = 194	13 (8–19)^*^ n = 157	13 (7–18)^†^ *n* = 54
Treatment on the intensive care unit	21.1% (41/194)	29.7% (47/158)	61.1% (33/54)^†^^‡^
Death	11.6% (22/189)	11.0% (17/154)	24.1% (13/54)^†^
**90 days follow up**			
Modified Rankin Scale score, median (Q1–Q3)	2 (1–5) *n* = 183	4 (3–6)^*^ n = 143	5 (4–6)^†^^‡^ *n* = 51
Good outcome (mRS 0–2)	54.1% (99/183)	20.3% (29/143)^*^	7.8% (4/51)^†^
Poor outcome (mRS 5–6)	25.7% (47/183)	42.7% (61/143)^*^	72.5% (37/51)^†^^‡^
Death	22.4% (41/183)	28.7% (41/143)	41.2% (21/51)^†^

*NIHSS, National Institutes of Health Stroke Scale; mRS, modified Rankin Scale*.

* *Indicates a significant difference between patients with an infection without sepsis and controls (p < 0.05 after Bonferroni-Holm adjustment for multiple comparisons)*.

† *Indicates a significant difference between sepsis patients and controls (p < 0.05 after Bonferroni-Holm adjustment for multiple comparisons)*.

‡ *indicates a significant difference between sepsis patients and patients with an infection without sepsis (p < 0.05 after Bonferroni-Holm adjustment for multiple comparisons)*.

The secondary outcome measures also demonstrated a significantly worse outcome in patients with sepsis compared to controls (median NIHSS at 24 h: 16 vs. 5; median NIHSS at discharge: 11 vs. 2; median length of stay 13 vs. 7 days; median mRS at 90 days: 5 vs. 2; frequency of mRS 0-2: 7.8 vs. 54.1%). Compared to patients with an infection without sepsis, the following secondary outcome measures showed a significantly worse outcome of patients with sepsis (median NIHSS at 24 h: 16 vs. 11; median NIHSS at discharge: 11 vs. 6; median mRS at 90 days: 5 vs. 4).

Clinical outcome was also analyzed in modified cohorts that included patients with sepsis and infection without sepsis, respectively, based on the SOFA score calculated without the CNS subcategory. This modified cohort of patients with sepsis included 51 patients, and the modified cohort of patients with an infection without sepsis 161 patients. The results were comparable to the analysis of the original cohort and are shown in Supplementary Table S3. Notably, poor outcome at 90 days was even more frequent in the modified sepsis cohort compared to controls and to the modified infection cohort than in the original cohort (80.9% vs. 25.7 and 40.8%, respectively). Further, in contrast to the original cohorts, mortality at 90 days in patients with sepsis was not only significantly higher than in controls, but also compared to patients with an infection without sepsis (53.2 vs. 22.4 and 25.2%, respectively). A sensitivity analysis of clinical outcome parameters after exclusion of 4 patients with comfort measures only agreements did not yield relevant differences compared to the analysis of the complete cohort. The results are shown in Supplementary Table S4.

Clinical outcome was further analyzed in patients with sepsis compared to propensity score matched cohorts of controls and patients with infection without sepsis. Five patients with sepsis had to be excluded for that analysis because of partially missing data for covariates of the propensity score and 3 further patients for partially missing outcome data, leaving 46 patients with sepsis for this analysis. A poor outcome at 90 days was more common in patients with sepsis compared to matched controls and to matched patients with an infection without sepsis (69.6 vs. 26.1 and 47.8%, respectively). The adjusted odds ratio (95% confidence interval) for a poor outcome was 9.0 (2.9–28.0) for patients with sepsis vs. controls, and 2.8 (1.1–6.9) for patients with sepsis versus patients with an infection without sepsis. Further results of the propensity score matched cohorts are reported in detail in Supplementary Table S5.

## Discussion

In this analysis of 406 patients who underwent endovascular therapy for LVOS, sepsis was detected in 13.3% with a median onset of 2 days after admission. The outcome at 90 days was poor (bedridden or dead) in 72.5% of patients with sepsis compared to 25.7% in control patients and to 42.7% in patients with an infection without sepsis.

A previous study observed sepsis in 12.6% of patients admitted to a neurological intensive care unit for ischemic or hemorrhagic stroke (5). While this frequency is numerically similar to our dataset, the results cannot be compared directly as the previous study analyzed a different category of patients and used a now outdated sepsis definition. The same holds true for the clinical outcome: An unfavorable outcome with high mortality was observed in sepsis patients in that study, but the control group also had a comparably poor outcome.

In our cohort, there were no relevant differences between controls and patients with sepsis regarding demographics, medical history as well as clinical, imaging and treatment characteristics at baseline, which could point to specific risk factors for sepsis in this cohort. While arterial hypertension was more prevalent in sepsis patients, the Charlson comorbidity index as a general measure of comorbidity was not significantly different between groups.

Pneumonia was the source of sepsis in the majority of patients (85.2 and 75.5% according to modified CDC criteria, respectively). Accordingly, the main risk factors for stroke-associated pneumonia, dysphagia with risk of aspiration and mechanical ventilation, were significantly more common in patients with sepsis. The overall frequency of pneumonia in our cohort (33.2 and 25.9% according to modified CDC criteria, respectively) was high compared to the frequency described for a general stroke population (12%) (3). This may be explained by the fact that all patients in our cohort had EVT in general anesthesia, which is associated with a higher risk of pneumonia. Indeed, a *post-hoc* analysis of the SWIFT PRIME thrombectomy trial demonstrated a similar frequency of pneumonia after EVT in general anesthesia as in our cohort (34.4%) (18). The overall infection rate including patients with and without sepsis was rather high (52.2%) in our cohort compared to the rates reported for general stroke populations (24–36%) and intensive care stroke patients (38–52%) (1). Possible reasons are that LVOS patients share more characteristics with intensive care patients than with general stroke patients, and that the diagnostic criteria for infections were liberal in our study. Application of strict operational criteria of infections leads to lower rate of infections, as reported above for pneumonia. Accordingly, the frequency of sepsis in a general stroke population including non-LVOS would be expected to be lower than in our cohort.

Pathophysiologically, there are several mechanisms making stroke patients prone to infections and consecutively sepsis. On the one hand, stroke patients often have an increased exposure to microbiological pathogens: Dysphagia and disorders of consciousness with impaired airway protection reflexes, and mechanical ventilation are risk factors for pneumonia (18, 19). Urinary catheters that are frequently used in severely affected stroke patients predispose to urinary tract infections. On the other hand, stroke affects the immune system as a host-intrinsic risk factor for infections (2, 19–21). Within hafter stroke, there is a systemic immunodepression, in particular a depression of CD4^+^ T-lymphocytes as well as reduced proinflammatory and increased anti-inflammatory cytokines, mainly driven by an excessive activation of the autonomic nervous system, increasing the susceptibility for infections (19, 20, 22, 23). In response to an infection, a host inflammatory response is initiated – in the case of sepsis, this response becomes dysregulated on the basis of both proinflammatory and anti-inflammatory mechanisms and compromises organ function (24, 25). Moreover, stroke-associated infections are suspected to trigger an autoimmune response against brain antigens, which may explain the poor outcome in these patients (2, 26). In stroke patients with sepsis, this phenomenon might be particularly important for clinical outcome given the central role of the dysregulated immune response in sepsis.

The most frequently affected organ systems in patients with sepsis were respiration, the cardiovascular system and renal function, consistent with pneumogenic sepsis. In a subset of patients without sepsis, the analysis of SOFA subcategories revealed relevant organ dysfunction, for example respiratory failure in 5.3% of patients with infection without sepsis. In these patients, the total SOFA score did not show an increase of ≥2 compared to admission, explaining why they were not classified as sepsis cases. A worsening of CNS function was often observed in sepsis patients, which might have been either caused by septic encephalopathy, by the stroke itself, mechanical ventilation with consecutive sedation, or other stroke-related complications. This might hamper the applicability of the SOFA score to diagnose sepsis in stroke patients. Indeed, we observed that 16.7% of patients with sepsis no longer fulfilled the Sepsis-3 definition if the SOFA score was calculated without the CNS subcategory. This indicates that the diagnosis of sepsis was based on a worsening of the neurological status in these patients. Thus, clinical reasoning is needed in practice to determine if worsening of the neurological status is due to septic encephalopathy, the stroke itself or other stroke-related complications.

The qSOFA score, a resource-efficient screening tool for sepsis, demonstrated very good sensitivity, but only mediocre specificity for the diagnosis of sepsis in our cohort. An analysis of the qSOFA subcategories revealed that the rather low overall specificity was due to low specificities of the mental status and respiratory rate categories. The drawbacks of using the neurological status for diagnosis of sepsis in stroke patients are the same as discussed above for the SOFA score. The respiratory rate subcriterion was often met in all 3 groups (60.2 to 81.2%), indicating that tachypnea occurred not only due to infection, but also due to the stroke itself, as disturbances of respiratory patterns with tachypnea are frequently observed in stroke patients (27, 28). Accordingly, tachycardia was frequently observed in all patients, which may rather reflect stroke-associated cardiovascular autonomic dysfunction than a clinical sign of infection or sepsis in these patients (29). Thus, our data confirm that the qSOFA score may be a helpful screening tool that should not be used without confirmatory tests, such as the regular SOFA score (6).

Patients with sepsis had a significantly worse clinical outcome than control patients without infection and patients with an infection but without sepsis. The most drastic difference was observed for a poor outcome (mRS five or six), but secondary outcome measures also showed a worse outcome in sepsis patients after adjustment for possible confounders. Notably, patients with sepsis had a significantly higher NIHSS at 24 h than controls and patients with an infection without sepsis. This difference cannot be attributed to septic encephalopathy in most patients, as the median onset of sepsis was 2 days after admission. If the NIHSS at 24 h was taken into account in multivariable analyses instead of the NIHSS at admission, sepsis was still an independent predictor of a poor outcome, but with a smaller effect size. Thus, stroke severity partly mediated the poor outcome observed in sepsis patients. An analysis restricted to sepsis patients in whom the diagnosis of sepsis was based on the dysfunction of organs other than the CNS also indicated that the association of sepsis with poor outcome was independent of factors affecting CNS function, such as stroke severity or mechanical ventilation with sedation.

The prevention, early diagnosis and effective treatment of sepsis might improve outcome after ET for LVOS. As the majority of cases were caused by pneumonia, preventative measures for pneumonia, such as swallowing assessments and therapy, oral hygiene measures and possibly the usage of conscious sedation for ET, may reduce the frequency of sepsis as well (18, 30–32). Previous trials showed that prophylactic antibiotic therapy does not improve outcome in stroke patients (33, 34). Thus, diagnostic criteria for infections, such as the SOFA score, should be applied thoroughly before antibiotic therapy is initiated. Collecting SOFA scores daily on a routine basis in patients with severe stroke would help with a timely diagnosis and might be a measure worthwhile exploring in future prospective studies.

This study has several limitations: the single center design, retrospective data collection and thus partly missing data, and that a special subgroup of stroke patients (LVOS undergoing ET) was studied, which limits the applicability of the results to a general population of stroke patients. A major limitation is that the SOFA score and thus the diagnosis of sepsis in stroke patients depended on stroke severity and other factors, such as mechanical ventilation. This might have affected the analysis of clinical outcome due to selection bias. Further, even though the same treatment guidelines were in use for all patients, the treatment might have deviated from the guidelines in some cases causing heterogeneity of treatment. Since we could not properly control for this, this is a potential source of omitted variable bias.

## Conclusions

Sepsis frequently occurs in patients with LVOS undergoing ET and may be associated with poor clinical outcome. More studies are needed to determine specific risk factors and measures for early recognition to reduce the possibly negative impact of sepsis on the outcome after LVOS.

## Data Availability Statement

The raw data supporting the conclusions of this article will be made available by the authors, without undue reservation.

## Ethics Statement

The studies involving human participants were reviewed and approved by the Ethics Committee of the Ludwig-Maximilian University Munich, Germany, and the Ethics Committee of the University of Bonn, Germany. The patients/participants provided their written informed consent to participate in this study.

## GSR-ET Investigators

A. Alegiani, J. Berrouschot, T. Boeckh-Behrens, G. Bohner, A. Bormann, M. Braun, M. Dichgans, F. Dorn, B. Eckert, U. Ernemann, J. Fiehler, C. Gerloff, K. Gröschel, GF. Hamann, KH. Henn, F. Keil, L. Kellert, C. Kraemer, A. Ludolph, CH. Nolte, M. Petersen, GC. Petzold, W. Pfeilschifter, S. Poli, J. Röther, E. Siebert, F. Stögbauer, G. Thomalla, S. Tiedt, C. Trumm, T. Uphaus, S. Wunderlich, S. Zweynert.

## Author Contributions

SS and GP conceptualized and designed the study and drafted the manuscript. All authors acquired and analyzed the data and approved the final version. All authors contributed to the article and approved the submitted version.

## Conflict of Interest

The authors declare that the research was conducted in the absence of any commercial or financial relationships that could be construed as a potential conflict of interest.

## Publisher's Note

All claims expressed in this article are solely those of the authors and do not necessarily represent those of their affiliated organizations, or those of the publisher, the editors and the reviewers. Any product that may be evaluated in this article, or claim that may be made by its manufacturer, is not guaranteed or endorsed by the publisher.
