# Supplementary material for: Sepsis in Patients With Large Vessel Occlusion Stroke–Clinical Characteristics and Outcome
Source: Front Neurol. 2022 Jul 12;13:902809. doi: 10.3389/fneur.2022.902809 (PMC9315268; doi:10.3389/fneur.2022.902809)
Supplement: Supplementary file 1 [file Data_Sheet_1.DOCX]

**DATA SUPPLEMENT**

**Sepsis in patients with large vessel occlusion stroke – clinical characteristics and outcome**

Sebastian Stösser, Julia Isakeit, Felix J. Bode, Christian Bode, Gabor C. Petzold; on behalf of the GSR-ET study group.

Corresponding Author:

Dr. Sebastian Stösser, Department of Neurology, Division of Vascular Neurology, University Hospital Bonn, Venusberg-Campus 1. 53127 Bonn, Germany
E-Mail: sebastian.stoesser@ukbonn.de

**Supplementary Figure 1.** Study flow chart





**Supplementary Figure 2.** Sequential Organ Failure Assessment (SOFA) scores of controls, patients with infection without sepsis and patients with sepsis over time. A) Total SOFA scores, B)-G) Scores in SOFA subcategories: B) Central nervous system, C) Respiration, D) Cardiovascular, E) Renal, F) Coagulation, G) Liver. “d0” represents the day of onset of infection, “d-2” and “d-1” the two days before, and “d+1” the day after.

**

**

**Supplementary Table 1.** Laboratory values, vital signs and Systemic Inflammatory Response Syndrome (SIRS) criteria of controls, patients with an infection without sepsis and patients with sepsis. The worst value of each individual patient within the period of data collection was included in the analysis (for patients with infection or sepsis from two days before to one day after the onset of infection; for controls with the first four days after admission).

|  | Controls  median (Q1-Q3) | Infection without sepsis  median (Q1-Q3) | Sepsis median (Q1-Q3) |
| --- | --- | --- | --- |
| Laboratory data (peak value) |  |  |  |
| Leukocytes, 10^9^/l | 10.0 (8.1-12.5) | 11.4 (9.3-14.7)* | 13.9 (11.1-17.5)†‡ |
| Procalcitonin, µg/l | 0.070 (0.040-0.140) | 0.125 (0.070-0.403)* | 0.225 (0.105-0.868)† |
| C-reactive protein, mg/l | 9.3 (2.9-26.2) | 45.9 (15.2-96.1)* | 25.9 (9.4-78.9)† |
| Lactate, mmol/l | 1.7 (1.3-2.1) | 1.7 (1.4-2.2) | 2.0 (1.6-2.6)† |
| Creatinine, mg/dl | 0.90 (0.75-1.10) | 0.93 (0.75-1.14) | 1.00 (0.74-1.30) |
| Urea, mg/dl | 31.4 (22.6-47.3) | 36.5 (31.2-54.0)* | 46.0 (30.6-62.0)† |
| Bilirubin, mg/dl | 0.57 (0.46-0.72) | 0.75 (0.44-1.05) | 0.63 (0.44-0.85) |
| Platelet count, G/l | 196.5 (160.5-249.0) | 194.5 (164.0-235.3) | 180.0 (145.8-230.0) |
| INR | 1.1 (1.0-1.1) | 1.1 (1.0-1.2)* | 1.1 (1.0-1.2)† |
| Vital signs (peak value) |  |  |  |
| Body temperature, °C | 37.5 (37.2-37.9) | 38.0 (37.7-38.4)* | 38.2 (37.7-38.7)† |
| Heart rate, per minute | 106 (92-120) | 105 (90-121) | 125 (107-149)†‡ |
| Respiratory rate,   per minute | 26 (23-28) | 27 (24-31)* | 29 (26-35)†‡ |
| SIRS criteria positive, % (n/N) | 68.0% (132/194) | 78.3% (123/157)* | 96.3% (52/54)†‡ |

INR = International normalized ratio, SIRS = Systemic Inflammatory Response Syndrome

‘*’ indicates a significant difference between patients with an infection without sepsis and controls (p<0.05 after Bonferroni adjustment for multiple comparisons).

‘†’ indicates a significant difference between sepsis patients and controls (p<0.05 after Bonferroni adjustment for multiple comparisons).

‘‡’ indicates a significant difference between sepsis patients and patients with an infection without sepsis (p<0.05 after Bonferroni adjustment for multiple comparisons).

**Supplementary Table 2.** Quick SOFA (qSOFA) scores in controls, patients with infection without sepsis and patients with sepsis

|  | Controls  % (n/N) | Infection without sepsis  % (n/N) | Sepsis  % (n/N) | Sensitivity for the diagnosis of sepsis | Specificity for the diagnosis of sepsis |
| --- | --- | --- | --- | --- | --- |
| qSOFA positive at diagnosis of infection | 37.2% (55/148) | 58.5% (86/147)* | 86.8% (46/53)†‡ | 86.8% | 52.2% |
| qSOFA subcategories positive at diagnosis of infection |  |  |  |  |  |
| Mental status | 45.4% (59/130) | 82.3% (93/113)* | 94.3% (50/53)†‡ | 94.3% | 37.5% |
| Respiratory rate | 60.2% (80/133) | 81.2% (95/117)* | 71.7% (38/53) | 71.7% | 30.0% |
| Systolic blood pressure | 26.4% (39/148) | 22.4% (33/147) | 49.1% (26/53)†‡ | 49.1% | 75.6% |

qSOFA = quick Sequential Organ Failure Assessment

‘*’ indicates a significant difference between patients with an infection without sepsis and controls (p<0.05 after Bonferroni adjustment for multiple comparisons).

‘†’ indicates a significant difference between sepsis patients and controls (p<0.05 after Bonferroni adjustment for multiple comparisons).

‘‡’ indicates a significant difference between sepsis patients and patients with an infection without sepsis (p<0.05 after Bonferroni adjustment for multiple comparisons).

**Supplementary Table 3.** Clinical outcome of controls, patients with an infection without sepsis and patients with sepsis. Allocation to groups was based on a modified SOFA score calculated without the CNS subcategory.

|  | Controls  % (n/N) | Infection without sepsis  % (n/N) | Sepsis  % (n/N) |
| --- | --- | --- | --- |
| **24 hours follow up** |  |  |  |
| NIHSS, median (Q1-Q3) n, available data | 5 (3-10)  n=162 | 11 (7-16)*  n=151 | 17 (13-21)†‡  n=40 |
| Any intracranial hemorrhage | 4.7% (9/192) | 8.2% (13/159) | 11.8% (6/51) |
| Symptomatic intracranial hemorrhage | 1.1% (2/190) | 2.5% (4/159) | 2.0% (1/49) |
| **Discharge follow up** |  |  |  |
| NIHSS, median (Q1-Q3) | 2 (0-5)  n=141 | 7 (2-12)*  n=112 | 11 (6-19)†‡  n=26 |
| Length of stay, d, median (Q1-Q3) | 7 (4-12)  n=194 | 13 (8-19)* n=160 | 13 (7-19)†  n=51 |
| Treatment on the intensive care unit | 21.1% (41/194) | 28.0% (45/161) | 68.6% (35/51)†‡ |
| Death | 11.6% (22/189) | 10.8% (17/157) | 25.5% (13/51) |
| **90 days follow up** |  |  |  |
| Modified Rankin Scale score, median (Q1-Q3) | 2 (1-5)  n=183 | 4 (3-6)*  n=147 | 6 (5-6)†‡  n=47 |
| Good outcome (mRS 0-2) | 54.1% (99/183) | 20.4% (30/147)* | 6.4% (3/47)† |
| Poor outcome (mRS 5-6) | 25.7% (47/183) | 40.8% (60/147)* | 80.9% (38/47)†‡ |
| Death | 22.4% (41/183) | 25.2% (37/147) | 53.2% (25/47)†‡ |

NIHSS = National Institutes of Health Stroke Scale, mRS = modified Rankin Scale

‘*’ indicates a significant difference between patients with an infection without sepsis and controls (p<0.05 after Bonferroni-Holm adjustment for multiple comparisons).

‘†’ indicates a significant difference between sepsis patients and controls (p<0.05 after Bonferroni-Holm adjustment for multiple comparisons).

‘‡’ indicates a significant difference between sepsis patients and patients with an infection without sepsis (p<0.05 after Bonferroni-Holm adjustment for multiple comparisons).

**Supplementary Table 4.** Sensitivity analysis of clinical outcome of controls, patients with an infection without sepsis and patients with sepsis after exclusion of patients with comfort measures only agreement (n=4)

|  | Controls  % (n/N) | Infection without sepsis  % (n/N) | Sepsis  % (n/N) |
| --- | --- | --- | --- |
| **24 hours follow up** |  |  |  |
| NIHSS, median (Q1-Q3) n, available data | 5 (3-10) n=162 | 11 (7-16)*  n=145 | 16 (12-20)†‡  n=42 |
| Any intracranial hemorrhage | 4.7% (9/192) | 8.5% (13/153) | 11.3% (6/53) |
| Symptomatic intracranial hemorrhage | 1.1% (2/190) | 2.6% (4/153) | 2.0% (1/51) |
| **Discharge follow up** |  |  |  |
| NIHSS, median (Q1-Q3) | 2 (0-5)  n=141 | 6 (2-12)*  n=110 | 11 (6-17)†‡  n=27 |
| Length of stay, d, median (Q1-Q3) | 7 (4-12)  n=194 | 13 (8-19)*  n=154 | 13 (7-18)†  n=51 |
| Treatment on the intensive care unit | 21.1% (41/194) | 29.7% (46/155) | 60.4% (32/53)†‡ |
| Death | 11.6% (22/189) | 9.9% (15/151) | 22.6% (12/53) |
| **90 days follow up** |  |  |  |
| Modified Rankin Scale score, median (Q1-Q3) | 2 (1-5)  n=183 | 4 (3-6)* n=140 | 5 (4-6)†‡  n=50 |
| Good outcome (mRS 0-2) | 54.1% (99/183) | 20.7% (29/140)* | 8.0% (4/50)† |
| Poor outcome (mRS 5-6) | 25.7% (47/183) | 42.1% (59/140)* | 72.0% (36/50)†‡ |
| Death | 22.4% (41/183) | 27.9% (39/140) | 40.0% (20/50) |

NIHSS = National Institutes of Health Stroke Scale, mRS = modified Rankin Scale

‘*’ indicates a significant difference between patients with an infection without sepsis and controls (p<0.05 after Bonferroni-Holm adjustment for multiple comparisons).

‘†’ indicates a significant difference between sepsis patients and controls (p<0.05 after Bonferroni-Holm adjustment for multiple comparisons).

‘‡’ indicates a significant difference between sepsis patients and patients with an infection without sepsis (p<0.05 after Bonferroni-Holm adjustment for multiple comparisons).

**Supplementary Table 5.** Clinical outcome of patients with sepsis compared to propensity score matched cohorts of patients with an infection without sepsis and controls.

|  | Controls  % (n/N) | Infection without sepsis  % (n/N) | Sepsis  % (n/N) |
| --- | --- | --- | --- |
| **24 hours follow up** |  |  |  |
| NIHSS, median (Q1-Q3) n, data available | 5 (3-12)  n=46 | 11 (7-16) n=46 | 16 (12-19) †‡ n=46 |
| Any intracranial hemorrhage | 8.7% (4/46) | 4.4% (2/46) | 13.0% (6/46) |
| Symptomatic intracranial hemorrhage | 2.3% (1/46) | 4.4% (2/46) | 2.3% (1/46) |
| **Discharge follow up** |  |  |  |
| NIHSS, median (Q1-Q3) | 2 (1-4) n=46 | 6 (3-12) n=46 | 11 (6-17)† n=46 |
| Length of stay, d, median (Q1-Q3) | 8.5 (5-13) n=46 | 14 (8-21) n=46 | 12 (6-17)† n=46 |
| Death | 15.2% (7/46) | 21.7% (10/46) | 21.7% (10/46) |
| **90 days follow up** |  |  |  |
| Modified Rankin Scale score, median (Q1-Q3) | 3 (1-5) n=46 | 4 (3-6) n=46 | 5 (4-6)† n=46 |
| Good outcome (mRS 0-2) | 45.7% (21/46) | 17.4% (8/46) | 8.7% (4/46)† |
| Poor outcome (mRS 5-6) | 26.1% (12/46) | 47.8% (22/46) | 69.6% (32/46) †‡ |
| Death | 23.9% (11/46) | 37.0% (17/46) | 39.1% (18/46) |

NIHSS = National Institutes of Health Stroke Scale, mRS = modified Rankin Scale

‘†’ indicates a significant difference between sepsis patients and controls (p<0.05 after Bonferroni-Holm adjustment for multiple comparisons).

‘‡’ indicates a significant difference between sepsis patients and patients with an infection without sepsis (p<0.05 after Bonferroni-Holm adjustment for multiple comparisons).

**Supplementary Methods**

A summary of the standard operating procedures for diagnosis and treatment of infections at our institution is given below in note form. The following manuals were in use caring for the patients included in this study:

- Medical Manual Stroke Unit (“Ärztliches Manual Stroke Unit”), Department of Neurology, University Hospital Bonn, updated annually, most recent update 14.07.2021
- Recommendations for therapeutic and prophylactic use of anti-infectives (“Empfehlungen zur Anti-Infektiva-Therapie und -Prophylaxe"), Medicines Committee, Task force for anti-infectives, Antibiotic steward ship team, University Hospital Bonn, most recent update 2017
- Manual of the operative intensive care units (“Manual der operativen Intensivstationen”), Department of Anesthesiology and Intensive Care Medicine, University Hospital Bonn, most recent update July 2019

Instructions for clinicians caring for stroke patients:

After stroke, patients are prone to infections. Pneumonia and urinary tract infections are the most common ones. Actively ask patients for typical symptoms during ward rounds and pay attention to typical findings on the daily physical exams. If patients or nurses report symptoms typical for an infection or you have a suspicion of an infection, start the diagnostic work up as outlined below. Additionally, if a stroke patient has a body temperature >38.0°C, assess him for symptoms and signs of an infection. Even if you do not find any clinical diagnostic clues for an infection, obtain an urinalysis, a chest x-ray, and a laboratory test including blood count and c-reactive protein and/or procalcitonin.

Pneumonia

- Typical signs: new onset of purulent sputum, increased secretions, dyspnea, tachypnea, cough, typical signs on auscultation, worsening gas exchange
- If there are at least two clinical signs present, obtain a chest x-ray and a laboratory test including blood count and c-reactive protein and/or procalcitonin.
- If there are new or progressive infiltrates on chest x-ray, and systemic signs of an infection (fever >38°C, leukocytosis/leukopenia, elevated c-reactive protein or procalcitonin), pneumonia can be diagnosed.
- Obtain additional microbiological tests: microscopy/cultures of sputum or tracheal secretions, blood cultures (if body temperature ≥38.5°C and in all cases of hospital acquired pneumonia), and, if needed, tests for legionella infections, aspergillosis and tuberculosis.
- First-line antibiotic therapy for the most common clinical scenarios:
  - Stroke-associated pneumonia: ampicillin/sulbactam 3 g i.v. TID for 7 days
  - Stroke-associated pneumonia with a risk of pseudomonas infection or a severe course: piperacillin/tazobactam 4.5 g i.v. TID for 7 days
  - Pneumogenic sepsis: piperacillin/tazobactam with a loading dose of 4.5 g, then 18 g i.v. continuously per day + tobramycin 5-7 mg/kg i.v. QD (dosing dependent on renal function and serum levels)

Urinary tract infection

- Typical symptoms and signs: dysuria, alguria, pollakisuria, suprapubic tenderness, fever (>38°C), change of color or odor of urine
- If there is at least one symptom or sign present, obtain an urinalysis (dipstick, sediment) and a urine culture.
- If there is evidence of bacteriuria (urine culture with ≥10^5^ CFU/ml and ≥10^3^ CFU/ml for catheter urine, alternatively: high count of bacteria in urine sediment), and leukocyturia (≥20/µl in urine sediment), urinary tract infection can be diagnosed.
- Differentiate asymptomatic bacteriuria from an urinary tract infection by a lack of typical symptoms and leukocyturia.
- First-line antibiotic therapy for the most common clinical scenarios:
  - Uncomplicated urinary tract infection in women: cotrimoxazole 960 mg p.o. BID for 3 days
  - Complicated urinary tract infection: cefuroxime 500 mg p.o. BID for 7 days OR cefuroxime 1.5 g i.v. TID for 7 days
  - Pyelonephritis: piperacillin/tazobactam 4.5 g i.v. TID for 10 days
  - Urosepsis: piperacillin/tazobactam with a loading dose of 4.5 g, then 18 g i.v. continuously per day + tobramycin 5-7 mg/kg i.v. QD (dosing dependent on renal function and serum levels)

Sepsis

- Diagnosis based on an increase in total SOFA score ≥2
- Initial diagnostic and therapeutic procedures within the first hour:
  - Complete physical exam
  - Monitoring of EKG, blood pressure, temperature, oxygen saturation
  - Laboratory tests including lactate and coagulation tests
  - Microbiological diagnostic: blood cultures (2 pairs), microscopy/cultures of tracheal secretions and urine, and other secretions or swabs if needed. Obtain cultures before initiation of antibiotic therapy without delaying the start of antibiotic therapy.
  - Find the focus: chest x-ray, sonography, age of catheters, and, if needed, echocardiography
  - Administration of crystalloid fluids (30 ml/kg) for hypotension or lactate ≥ 4 mmol/l (within the first 3 hours) and noradrenaline if necessary. Goals of therapy: mean arterial pressure ≥ 65 mmHg, lactate < 2 mmol/l, urine output > 0.5 ml/kg
  - Establish a central venous catheter and an arterial catheter (or a catheter for arterial pulse-wave analysis in septic shock)
  - Administer broad-spectrum antibiotics according to the suspected focus (see above for the most common sources pneumonia and urinary tract infection)
  - Consider orotracheal intubation and mechanical ventilation in patients with pneumonia or impaired consciousness
- Follow-up diagnostic and therapeutic procedures within the first six hours:
  - Find the focus. If still unclear, computed tomography (head, thorax, abdomen). Evaluate catheters again.
  - Treat the focus (e.g. surgery consult)
  - In septic shock (mean arterial pressure < 65 mmHg, lactate > 2 mmol/l, need for increasing doses of noradrenaline): echocardiography and advanced hemodynamic monitoring (catheter for arterial pulse-wave analysis or pulmonary artery catheter). Optimize cardiac preload using crystalloid fluids. Add other vasopressors if needed (dobutamine, vasopressin, milrinone, adrenaline)
  - In pneumogenic sepsis, bronchoscopy to take specimen for microbiological testing
  - Serial monitoring of lactate
  - Broad microbiological/virological testing as needed, e.g. aspergillosis, clostridium difficile, influenza, agents of atypical pneumonia, analysis of cerebrospinal fluid including microscopy and cultures
  - Lung protective ventilation: tidal volume 6 ml/kg, maximal pressure <30 mbar, positive end-expiratory pressure ≥10 mbar, permissive hypercapnia if pH ≥7,25 and no contraindications
  - In suspected catheter-related blood stream infections, change catheters (if not done initially)
  - Consider renal replacement therapy in acute renal failure or lactic acidosis
  - Consider administration of hydrocortisone in refractory septic shock
  - In hyperglycemia, administer insulin (goal <180 mg/dl)

**Appendix**

List of GSR-ET Investigators: A Alegiani, J Berrouschot, T Boeckh-Behrens, G Bohner, A Bormann, M Braun, M Dichgans, F Dorn, B Eckert, U Ernemann, J Fiehler, C Gerloff, K Gröschel, GF Hamann, KH Henn, F Keil, L Kellert, C Kraemer, A. Ludolph, CH Nolte, M Petersen, GC Petzold, W Pfeilschifter, S Poli, J Röther, E Siebert, F Stögbauer, G Thomalla, S Tiedt, C Trumm, T Uphaus, S Wunderlich, S Zweynert.
